# Supplementary material for: Specific Mutations near the Amyloid Precursor Protein Cleavage Site Increase γ-Secretase Sensitivity and Modulate Amyloid-β Production
Source: Int J Mol Sci. 2023 Feb 16;24(4):3970. doi: 10.3390/ijms24043970 (PMC9959964; doi:10.3390/ijms24043970)
Supplement: Supplementary file 1 [file ijms-24-03970-s001.zip › ijms-2119571-supplementary.pdf]

Supplementary Figures

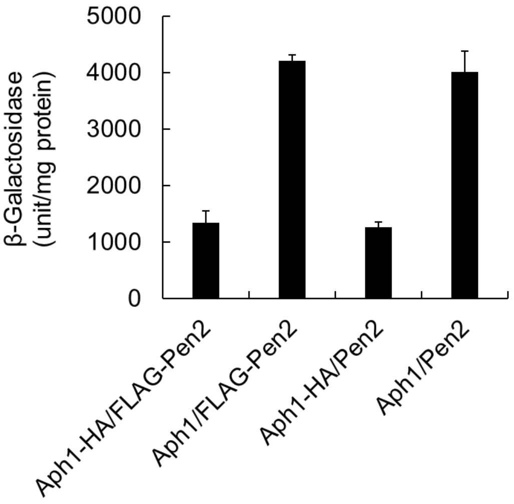

(a)

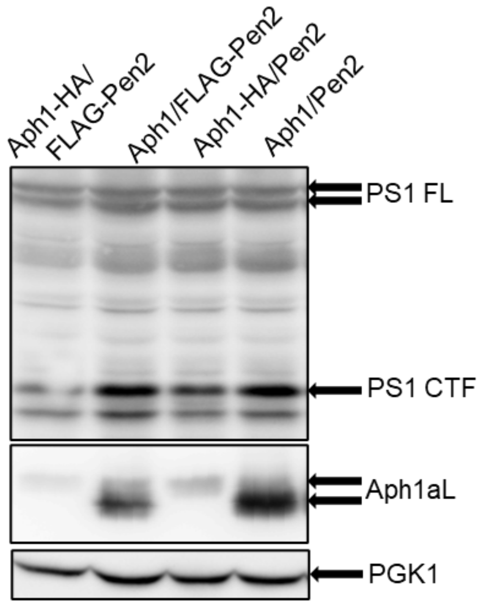

(b)

Supplementary Figure S1. Comparison of Pen2 and Aph-1aL with or without detection tags. γ-Secretase was reconstituted by PS1, NCT, FLAG-Pen2 or Pen2, and Aph-1aL-HA or Aph-1aL in yeast. **(a)** β-Galactosidase activity was assayed as in Figure 2. The carboxy terminal HA tag cause reduced β-galactosidase activity.; **(b)** Expression of PS1 and Aph-1aL. Cell lysates were analyzed using immunoblot analysis and antibodies against PS1 loop (GIL3) or Aph1-aL carboxyl fragment (245–265; BioLegend). We also detected PGK1 using a PGK1 antibody (Abcam) as control. The arrows indicate PS1 FL, PS1 CTF, Aph-1aL, and PGK1. The carboxy terminal HA tag reduced the amount of Aph1-aL and PS1CTF, which is the mature form of PS1 in the γ-secretase complex. Aph-1aL-HA migrate slowly comparing to Aph-1aL without tag.

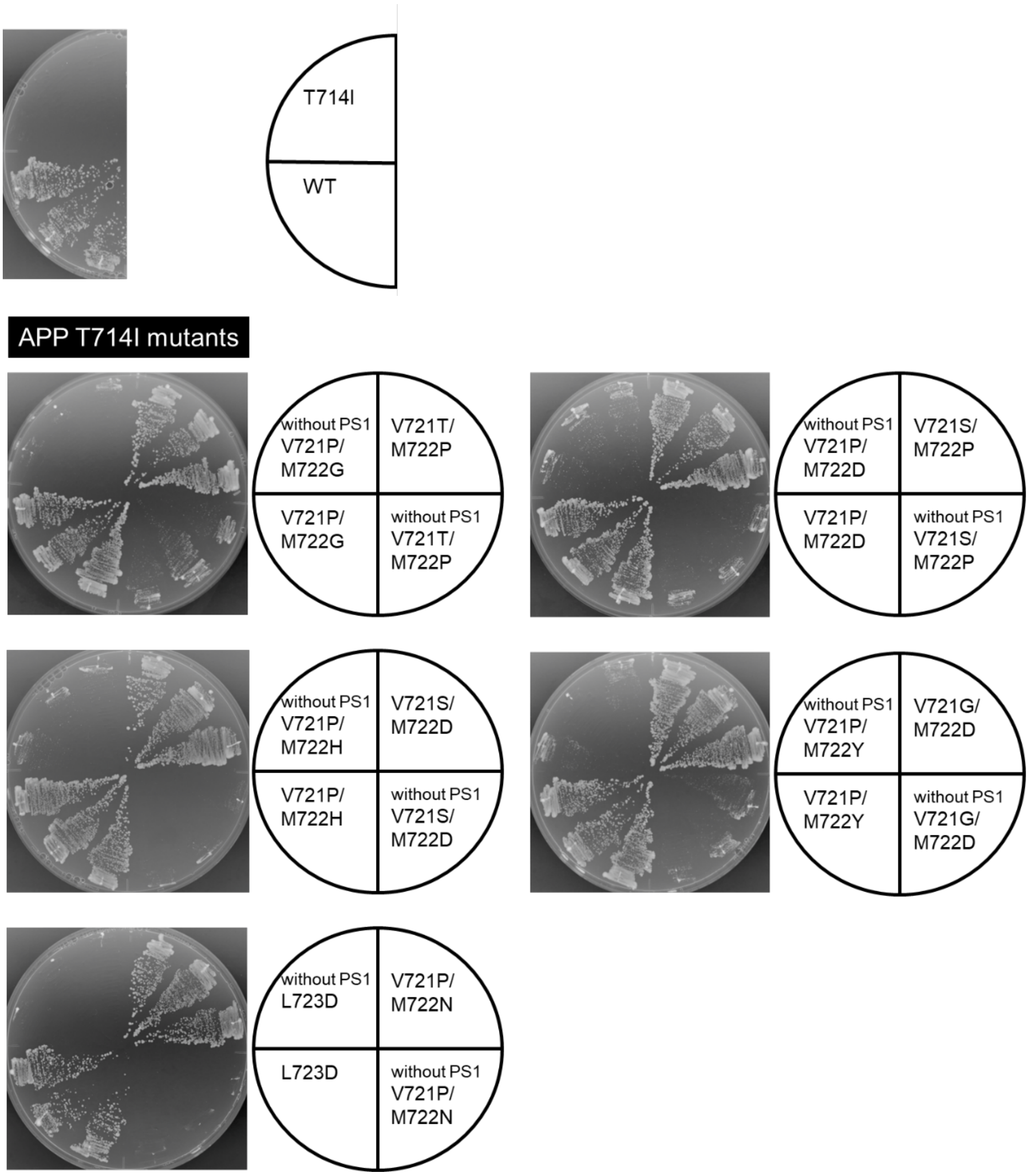

Supplementary Figure S2. Growth of secondary mutants that restored cleavage of the APP T714I FAD mutant. The growth of cells containing APP mutations was examined after incubation at 30°C for 3 days on SD-LWHUA media. All cells contained NCT, Aph-1aL-HA, and FLAG-Pen2 with or without PS1. Summary of growth evaluation is shown as Table 2.

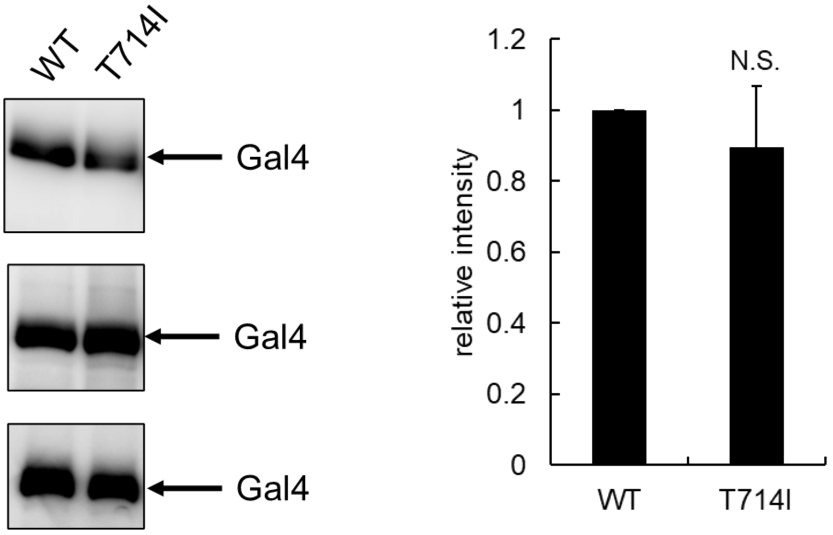

(a) (b)

Supplementary Figure S3. Expression of wild-type (WT) or T714I APPC55-Gal4p in yeast. WT or T714I APPC55-Gal4p was introduced into yeast with PS1, NCT, FLAG-Pen2, and Aph-1aL-HA. **(a)** Cell lysates from the APP WT or T714I mutant were analyzed using immunoblot analysis and an antibody against Gal4. The upper panel is the same blot as shown in Figure3b. The arrow indicates the position of Gal4.; **(b)** Relative intensity of APPC55-Gal4p was quantified. Three independent assays in **(a)** were quantified and shown with standard deviations. Statistical analyses were performed using the Student's *t* test.

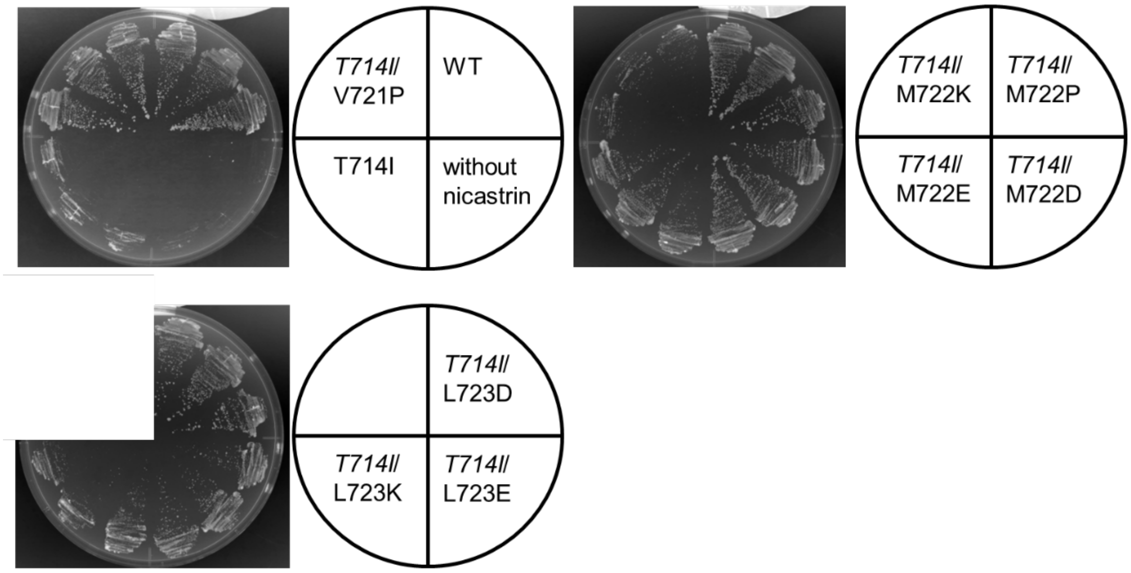

Supplementary Figure S4. Growth of the aspartate and glutamate mutations. The growth of cells containing WT or mutant APP (T714I, *T714I*/V721P, *T714I*/M722P, *T714I*/M722D, *T714I*/M722E, *T714I*/M722K, *T714I*/L723D, *T714I*/L723E and *T714I*/L723K) were examined after incubation at 30°C for 3 days on SD-LWHUA media. All cells contained PS1, Aph-1aL-HA, and FLAG-Pen2 with or without NCT.
